# Macrophages in close proximity to the vitreoretinal interface are potential biomarkers of inflammation during retinal vascular disease

**DOI:** 10.1186/s12974-022-02562-3

**Published:** 2022-08-08

**Authors:** Amrita Rajesh, Steven Droho, Jeremy A. Lavine

**Affiliations:** Department of Ophthalmology, Feinberg School of Medicine, Northwestern University, 240 E Huron St, McGaw M343, Chicago, IL 60611 USA

**Keywords:** Biomarkers, Diabetic retinopathy, Inflammation, Macrophage, Optical coherence tomography, Retinal vein occlusion, Vitreoretinal interface

## Abstract

**Background:**

Diabetic retinopathy and retinal vein occlusion are vision threatening retinal vascular diseases. Current first-line therapy targets the vascular component, but many patients are treatment-resistant due to unchecked inflammation. Non-invasive inflammatory imaging biomarkers are a significant unmet clinical need for patients. Imaging of macrophage-like cells on the surface of the retina using clinical optical coherence tomography (OCT) is an emerging field. These cells are increased in patients with retinal vascular disease, and could be a potential inflammatory biomarker. However, since OCT is limited by an axial resolution of 5–10 microns, the exact location and identity of these retinal cells is currently unknown.

**Methods:**

We performed OCT followed by confocal immunofluorescence in wild-type mice to identify macrophages within 5–10 microns of the vitreoretinal interface. Next, we used *Cx3cr1*^*CreER/*+^; *Rosa26*^*zsGreen/*+^ mice to fate map retinal surface macrophages. Using confocal immunofluorescence of retinal sections and flatmounts, we quantified IBA1^+^*Tmem119*^+^CD169^neg^ microglia, IBA1^+^*Tmem119*^neg^CD169^neg^ perivascular macrophages, and IBA1^+^*Tmem119*^neg^CD169^+^ vitreal hyalocytes. Finally, we modeled neuroinflammation with CCL2 treatment and characterized retinal surface macrophages using flow cytometry, OCT, and confocal immunofluorescence.

**Results:**

We were able to detect IBA1^+^ macrophages within 5–10 microns of the vitreoretinal interface in wild-type mice using OCT followed by confirmatory confocal immunofluorescence. Retinal surface macrophages were 83.5% GFP^+^ at Week 1 and 82.4% GFP^+^ at Week 4 using fate mapping mice. At steady state, these macrophages included 82% IBA1^+^*Tmem119*^+^CD169^neg^ microglia, 9% IBA1^+^*Tmem119*^neg^CD169^+^ vitreal hyalocytes, and 9% IBA1^+^*Tmem119*^neg^CD169^neg^ perivascular macrophages. After CCL2-driven neuroinflammation, many Ly6C^+^ cells were detectable on the retinal surface using OCT followed by confocal immunofluorescence.

**Conclusions:**

Macrophages within close proximity to the vitreoretinal interface are self-renewing cells, and predominantly microglia with minor populations of perivascular macrophages and vitreal hyalocytes at steady state. In the context of neuroinflammation, monocytes and monocyte-derived macrophages are a significant component of retinal surface macrophages. Human OCT-based imaging of retinal surface macrophages is a potential biomarker for inflammation during retinal vascular disease.

**Supplementary Information:**

The online version contains supplementary material available at 10.1186/s12974-022-02562-3.

## Introduction

Diabetic retinopathy (DR) and retinal vein occlusion (RVO) are common, vision threatening retinal diseases. Vision loss occurs through macular edema, macular ischemia, and complications of proliferative retinopathy including neovascularization, vitreous hemorrhage, and tractional retinal detachment. Current first-line therapy includes anti-vascular endothelial growth factor (VEGF) injections, which are safe and effective in treating macular edema and regress neovascularization [1–4]. However, 32–66% of patients [5] with diabetic macular edema (DME) and 46–72% of patients with macular edema from RVO [1] have persistent retinal thickening after 6 monthly intravitreal anti-VEGF injections. Therefore, treatment resistance is an unmet clinical need.

Although DR and RVO are retinal vascular diseases, inflammation plays a critical role. In mouse models of DR, classical monocytes promote DR progression [6], while microglia [7] and non-classical monocytes [8] inhibit DR. Similarly, classical monocytes are pro-inflammatory and non-classical monocytes protect endothelial cells from apoptosis in a mouse model of RVO [9]. Furthermore, intravitreal steroids are an effective therapy for both DME [10] and RVO [11], but are second line due to increased risk of glaucoma and cataract. These data highlight the importance of monocytes, macrophages, and their heterogeneity in DR and RVO-associated inflammation.

Due to the important role of inflammation in retinal vascular disease, inflammatory biomarkers are an important area of investigation. Using optical coherence tomography (OCT) and adaptive optics scanning laser ophthalmoscopy (AO-SLO), mobile, ramified macrophage-like cells are detectable on the surface of the retina in human patients [12, 13]. In mice, similar cells are *Cx3cr1*^+^, supporting a monocyte or macrophage lineage [14]. Clinically, macrophage-like cell numbers are increased in patients with proliferative DR (PDR) [15] and in patients with RVO, correlating with both ischemia and macular edema [16]. These findings suggest that macrophage-like cells could be an inflammatory biomarker in retinal vascular disease. However, because OCT has an axial resolution of 5–10 microns [17], while AO-OCT is far worse, the exact location of these cells and their identity remains unknown.

The goal of this study was to comprehensively characterize macrophage heterogeneity in close proximity to the vitreoretinal interface at both steady state and during inflammation in mice. The exact vitreoretinal interface is the apposition between the internal limiting membrane (ILM) of the retina, which is a basement membrane comprising type IV collagen, and the collagen II rich posterior cortex of the vitreous. However, since OCT has an axial resolution of 5–10 microns, and our goal is to determine the identity of macrophage-like cells on human OCT imaging, we need to consider cells and anatomic spaces within 5–10 microns of the vitreoretinal interface. Hyalocytes are resident immune cells which have previously been identified on the vitreous side of the ILM [18]. Within the retina, the nerve fiber layer exists just below the ILM and microglia are present in the retinal nerve fiber layer in addition to the inner and outer plexiform layers [19]. Finally, large retinal vessels traverse the retina through the retinal nerve fiber layer, just below the ILM. Along these vessels, a unique anatomical space exists, where perivascular macrophages reside, delineated by two membranes: the glia limitans superficialis on the parenchymal side and the glia limitans vascularis on the endothelial side [20]. Thus, potential macrophage-like cells on OCT imaging of the vitreoretinal interface in humans could include hyalocytes, microglia, perivascular macrophages, and infiltrating inflammatory cells, which are all within 5–10 microns of the true vitreoretinal interface.

Using OCT imaging, we identified cells within 5–10 microns of the vitreoretinal interface that corresponded to IBA1^+^ macrophages on confocal immunofluorescence. Using fate mapping mice, we found that these macrophages are self-renewing cells. Next, via confocal imaging of retinal sections, retinal flatmounts, and 3D reconstructions, we showed that macrophages near the vitreoretinal interface include 82% IBA1^+^*Tmem119*^+^CD169^neg^ microglia with 9% of both IBA1^+^*Tmem119*^neg^CD169^+^ vitreal hyalocytes and IBA1^+^*Tmem119*^neg^CD169^neg^ perivascular macrophages at steady state. In the context of CCL2-driven inflammation, Ly6C^+^ cells were detectable at the retinal surface by OCT imaging and confocal immunofluorescence. These data demonstrate that macrophages within 5–10 microns of the true vitreoretinal interface include predominantly microglia with minor populations of vitreal hyalocytes and perivascular macrophages at steady state. During inflammation, however, retinal surface macrophages include a large number of blood-derived inflammatory cells. Therefore, human imaging of macrophage-like cells is a potential inflammatory biomarker during retinal vascular disease.

## Methods

### Animals

Wild-type C57BL/6J (#000,664), *Cx3cr1*^*CreER*^ (#020,940), *Rosa26*^*zsGreen*^ (#007,906), and *Tmem119*^*GFP*^ (#031,823) mice were purchased from Jackson Labs (Bar Harbor, ME). Wild-type C57BL/6J mice were bred in-house and maintained in a pathogen-free barrier facility within Northwestern University’s Center for Comparative Medicine. *Cx3cr1*^*CreER/CreER*^ and *Rosa26*^*zsGreen/zsGreen*^ were crossed to generate *Cx3cr1*^*CreER/*+^; *Rosa26*^*zsGreen/*+^ for fate mapping (Mac^GFP^ mice). *Tmem119*^*GFP/GFP*^ and C57BL/6 J mice were bred to create *Tmem119*^*GFP/*+^ for experiments. The absence of the RD8 allele (*Crb1*^*−*^) and the correct genotype were confirmed by genotyping one complete litter from each breeding pair. Genotyping was performed by Transnetyx (Cordova, TN). All experiments were conducted in accordance with the ARVO Statement for the Use of Animals in Ophthalmic and Vision Research and were approved by the Northwestern University Institutional Animal Care and Use Committee. All experiments were performed on 10–12 week-old female mice unless otherwise stated.

### Optical coherence tomography (OCT)

OCT was performed on the Spectralis OCT2 system (Heidelberg Engineering, Heidelberg, Germany). Mice were anesthetized with a ketamine/xylazine cocktail, eyes were dilated, and meloxicam was administered for pain prophylaxis, as previously described [21]. A contact lens was placed on the surface of the eye (Cantor and Nissel, 3.2 mm diameter, 1.7 mm base curve, #90,642). The mouse was placed on the animal holder and the teeth were secured to the bite bar. The OCT2 system was focused upon the retinal nerve fiber bundles using infrared imaging. Infrared reflectance, OCT (detail scan, 25 averaged frames) and OCT-angiography (high resolution, 7 averaged frames) imaging were performed in posterior regions with unique vascular patterns to help identify regions of interest on immunofluorescence. In HEYEX 2 software (Heidelberg Engineering), an en face maximum intensity projection reconstruction directly above the vitreoretinal interface was performed to mimic the 0–3 micron slab for macrophage-like cells in human imaging [12, 15].

### Immunofluorescence imaging

Eyes were prepared as previously described [21]. Briefly, mice were killed, eyes were enucleated, and fixed in 1% paraformaldehyde (#15,713-S; Electron Microscopy Sciences, Hatfield, PA, USA) for 1 h at room temperature. For retinal flatmounts, retinas were dissected in 1X TBS (Tris-buffered saline) under a microscope. The retina was blocked in TBS + 5% Donkey Serum (S30, Sigma-Aldrich, St. Louis, MO) overnight at 4^0^C. Retinas were treated with primary antibodies (Table 1) overnight at 4^0^C and washed 5 times with TBS-T (TBS with 0.5% Tween-20, #00,777; Amresco, Solon, OH, USA). Next, retinas were treated with secondary antibodies overnight at 4^0^C (Table 1), washed 5 times with TBS-T, and mounted on HistoBond microscope slides (16,004–406, VWR; Batavia, IL, USA) in Immu-Mount (#9,990,402; ThermoFisher, Carlsbad, CA, USA).

**Table 1:** Immunofluorescence imaging antibodies

Antibody	Fluorophore	Dilution	Manufacturer, product number
Goat anti-mouse CD31	–	1:250	R&D Systems, AF3628
Rabbit anti-mouse IBA1	–	1:500	Wako, 019–19,741
Chicken anti-mouse GFP	–	1:6000	Abcam, ab13970
Rat anti-mouse ICAM2	–	1:500	BD Biosciences, 553,326
Rabbit anti-mouse TMEM119	–	1:1000	Synaptic Systems, 400 002
Rat anti-mouse CD169	–	1:200	Bio-Rad, MCA884
Rat anti-mouse Ly6C	–	1:1000	Abcam, ab54223
Rat anti-mouse CD206	–	1:200	Bio-Rad, MCA2235
Rabbit anti-Collagen IV	–	1:500	Abcam, 19,808
Donkey anti-goat (H + L)	Alexa Fluor 405	1:500	Invitrogen, A48259
Donkey anti-rabbit (H + L)	Alexa Fluor 647	1:500	Invitrogen, A31573
Donkey anti-chicken (H + L)	Alexa Fluor 488	1:500	Jackson ImmunoResearch, 703–545-155
Donkey anti-rat (H + L)	TRITC	1:500	Invitrogen, A18750

**Fig. 1:**
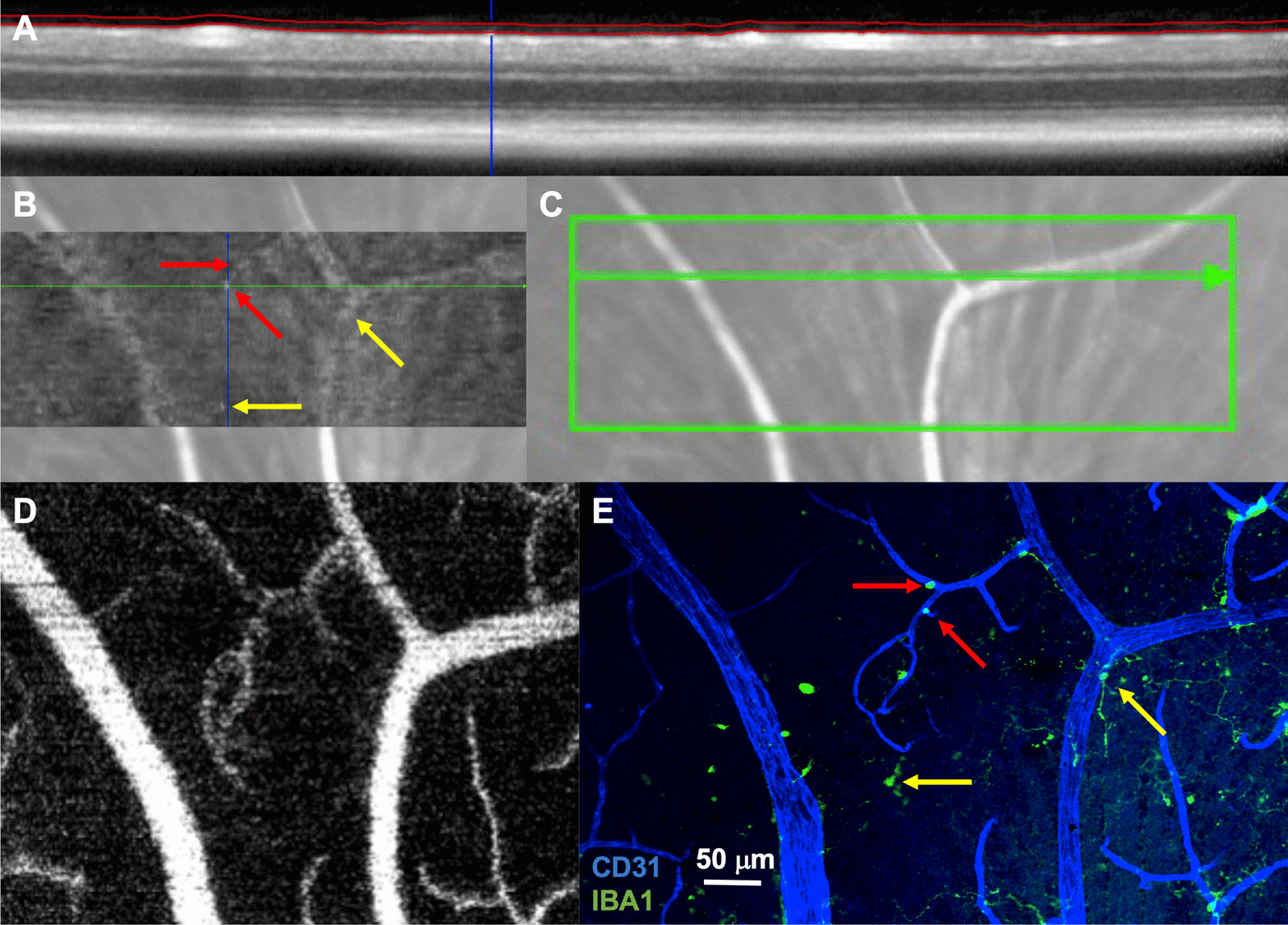
Macrophages are detectable at the retinal surface using OCT. **A** OCT B-scan showing segmentation of the vitreoretinal interface slab. **B** En face slab with cells highlighted using arrows. **C** Infrared reflectance showing the OCT scan location. **D** OCT-angiography image of part of the area from **C**. **E** Confocal immunofluorescence of the superficial retina (CD31 = blue, IBA1 = green). Matching ramified macrophages (indicated by yellow arrows) and possible perivascular macrophages (red arrows). Representative image from 5 independently imaged mice from a single litter

**Fig. 2:**
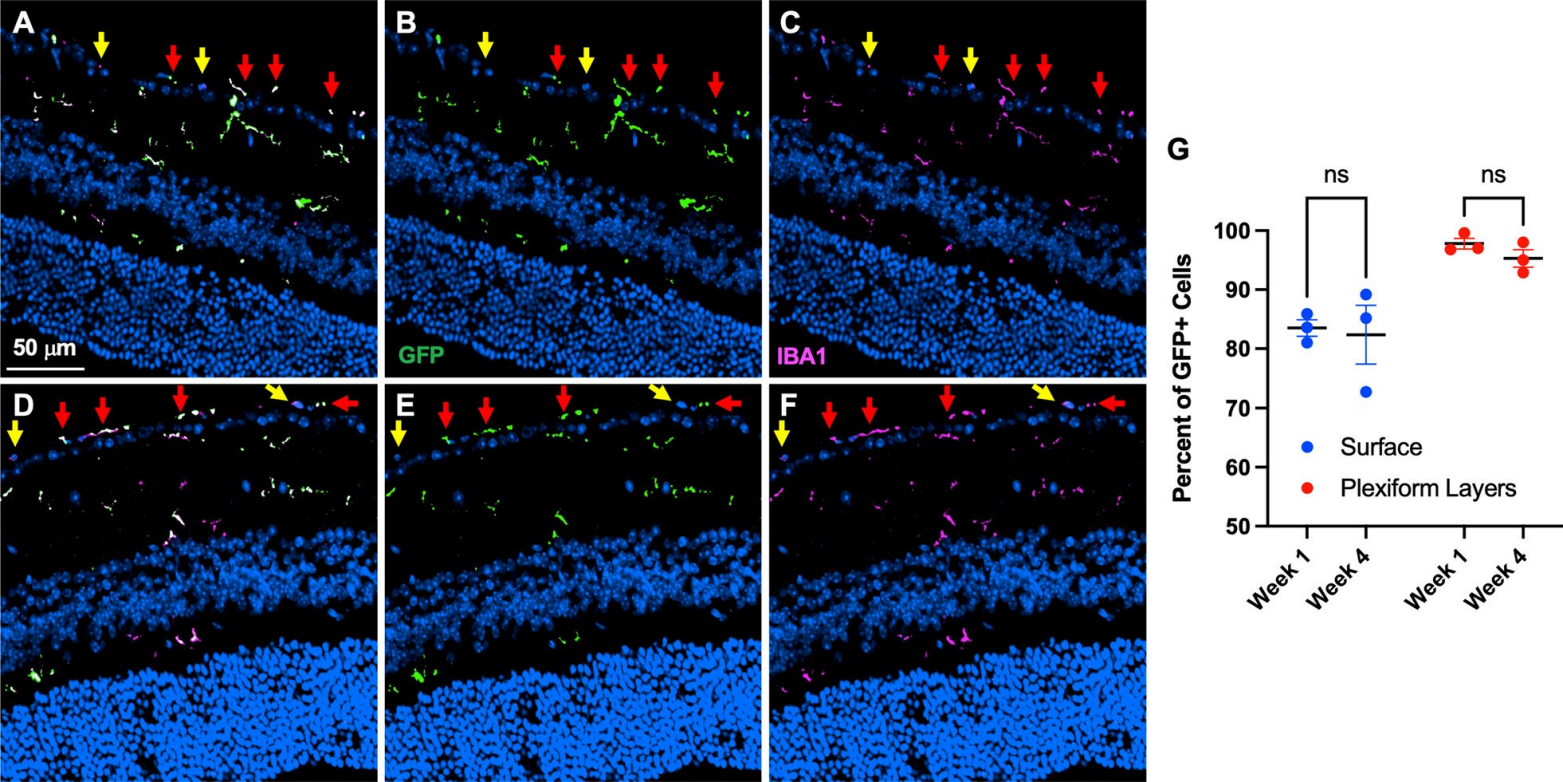
Macrophage fate mapping shows that retinal surface macrophages are self-renewing cells. Representative retina sections of Mac^GFP^ mice at 1 week (**A**–**C**) or 4 weeks (**D**–**F**) after tamoxifen administration. Channels are separated showing merged (**A**, **D**), DAPI and GFP (**B**, **E**), or DAPI and IBA1 (**C**, **F**) staining. Red arrows highlight GFP^+^ cells and yellow arrows highlighting GFP^neg^ cells. Surface macrophages are significantly less GFP^+^ than plexiform macrophages (**G**, *p* < 0.05, two-way ANOVA followed by Šídák's multiple comparisons test). No changes between Week 1 and Week 4 GFP^+^ cells were detected (**G**). *N* = 3 mice per group from 1 experiment. Two litters were consecutively treated with tamoxifen. One litter was killed at 1 week and the second litter was killed 1 month post-tamoxifen

**Fig. 3:**
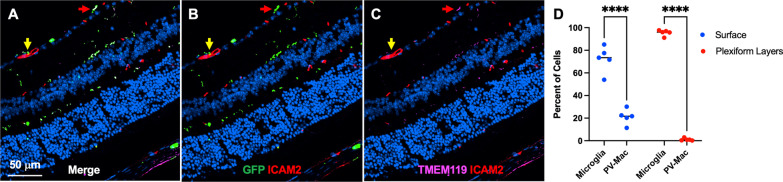
Surface macrophages comprised microglia and potentially perivascular macrophages. Representative merged (**A**), GFP and ICAM2 (**B**), and TMEM119 and ICAM2 (**C**) images of frozen sections from Mac^GFP^ mice. Arrows highlight TMEM119^+^ microglia (red) and TMEM119^neg^ perivascular macrophages (yellow). **D** At the retinal surface, macrophages were 72.3% GFP^+^TMEM119^+^ microglia and 21.2% GFP^+^TMEM119^neg^ICAM2^adjacent^ perivascular macrophages (PV-Mac), while plexiform layer macrophages were almost entirely microglia (****p < 0.001, two-way ANOVA followed by Šídák's multiple comparisons test). *N* = 5 mice from 2 separate experiments (1 litter each) where tamoxifen was given independently

For frozen sections, eyes were dissected in PBS to remove the conjunctiva and extraocular muscles after fixation. Next, two perpendicular incisions were made along the cornea to insert forceps and carefully remove the lens. The eye was then washed in PBS, 10%, 20%, and 30% sucrose (4097–04, J.T. Baker, Central Valley, PA, USA) solutions in PBS for 1 h each. Next, the eye was placed on a 15 mm x 15 mm x 15 mm vinyl specimen mold (4566, Sakura, CA, USA) with the optic nerve placed parallel to the bottom of the mold. The mold was filled with clear optimal cutting temperature compound (23-730-571, Fisher Healthcare, Pittsburg, PA) and then frozen. A cryostat was used to create 8-micron sections. Sections were stained by washing with PBS for 10 min, blocking for 1 h at room temperature in 5% donkey serum, and then stained identically to retinal flatmounts except PBS was substituted for TBS-T (Table 1). Immunofluorescence imaging was performed on a Nikon W1 Dual CAM Spinning Disk Microscope (Figs. 1, 2, 3, 5, 7, 8) using Nikon NIS Elements software. Five to ten sections were reviewed per mouse for Figs. 2, 3.

### 3D reconstructions

Images (1024 × 1024 pixels) were captured on a Nikon AXR confocal microscope (Melville, NY) using a 60 × oil objective with a 1.4 numerical aperture. Z-steps of 0.2 microns were used from the retinal surface to the intermediate vascular plexus. Imaris 9.8 software (Oxford Instruments, UK) was used to classify surfaces, eliminate noise in each channel, and create 3D reconstructions.

### Tamoxifen administration

Injections were prepared and performed as previously described [22]. Briefly, tamoxifen (T5648, Sigma-Aldrich) was dissolved in corn oil (C8267, Sigma-Aldrich) at 20 mg/ml and was stored for less than one week. Two intraperitoneal injections were performed 2 days apart in 6- to 8-week-old Mac^GFP^ mice. Mice were killed for immunofluorescence imaging of frozen sections 1 week or 4 weeks post-tamoxifen treatment.

### Intravitreal injections

Mice were anesthetized with ketamine/xylazine cocktail, eyes were dilated, and meloxicam was administered for pain prophylaxis as previously described [21]. Under a microscope, a 30-gauge needle was used to create an incision just posterior to the sclerocorneal junction. Next, 5 ng of CCL2 (479-JE-050/CF, R&D Systems Inc., Minneapolis, MN) in 1 µl of PBS, or sterile PBS vehicle control, were injected intravitreally using a Hamilton Syringe (7648-01, Hamilton, Reno, Nevada) on a 32-gauge needle (0.4 in. PT3, Hamilton). Eyes were covered in erythromycin ointment and the mice recovered from anesthesia on a heating pad. OCT, immunofluorescence imaging of retinal flatmounts, and/or flow cytometry were performed 24 h later.

### Flow cytometry of mouse retina

Eyes were enucleated and stored briefly in HBSS (14,025,076, Gibco, Carlsbad, CA). Eyes were dissected on a microscope to isolate retina alone. The retina was cut into at least four pieces and digested in Liberase TL for one hour at 37^0^C with shaking (200 RPM). Post digestion, retinal cells were treated identically to our previously published flow cytometry procedure [23]. Briefly, a single cell suspension was obtained and stained for innate immune cell markers (see Table 2). Samples were run on a FACSymphony A5-Laser Analyzer (Becton Dickinson, CA, USA). Experiments were performed on two separate days and combined.

**Table 2:** Flow cytometry antibodies

Antibody	Fluorophore	Manufacturer, product number
Fc block	–	BD Biosciences, 553,142
Aqua Live/Dead	AmCyan	ThermoFisher, 65–0866-14
CD45	BUV395	BD Biosciences, 564,279
CD64	PE	BD Biosciences, 139,304
CD11b	APC-Cy7	BD Biosciences, 557,657
Ly6G	PE-CF594	BD Biosciences, 562,700
B220	PE-CF594	BD Biosciences, 562,313
NK1.1	PE-CF594	BD Biosciences, 562,864
SiglecF	PE-CF594	BD Biosciences, 562,757
CD4	PE-CF594	BD Biosciences, 562,314
CD8	PE-CF594	BD Biosciences, 562,315
Ly6C	FITC	BD Biosciences, 561,085
Cx3Cr1	Alexa Fluor 647	Biolegend, 149,004
CD206	PE-Cy7	Biolegend, 141,720

### Statistical analysis

Statistical analyses were performed using GraphPad Prism 9.0.1 (GraphPad Software, San Diego, California USA). Normality was tested using the Shapiro–Wilk test. Differences between percent GFP^+^ cells in fate mapping studies were compared using two-way ANOVA followed by Šídák's multiple comparisons test (Figs. 2, 3). The number of microglia, perivascular macrophages, and hyalocytes were analyzed using repeated measures analysis of variance (ANOVA) followed by Tukey’s multiple comparisons test (Fig. 5). Flow cytometry data comparisons were made using two-tailed unpaired t tests with Welch’s correction (Fig. 6).

## Results

Macrophage-like cells are detectable on the surface of the retina in human patients using clinical OCT imaging [12, 15]. However, macrophages are heterogeneous, and the identity of these macrophages is unknown. Furthermore, since the axial resolution of OCT is limited to 5–10 microns, the exact location of these cells is unclear. We performed OCT in mice to determine if these same cells are detectable. OCT images were obtained, focusing upon the retinal surface, in wild-type mice. A thin section was demarcated at the vitreoretinal interface (red lines, Fig. 1A) and an en face map of this slab was generated (Fig. 1B). We identified protrusions of cell-like structures on the surface of the retina in the B-scan (blue line, Fig. 1A) and in the en face map (intersection of blue and green lines, Fig. 1B). Both infrared reflectance (Fig. 1C) and OCT-angiography (superficial vascular plexus, Fig. 1D) were used to identify the exact same region for confocal immunofluorescence. We corresponded CD31^+^ large vessels with large superficial vessels on OCT-angiography to ensure that flatmounts were imaged confocally within 5–10 microns of the vitreoretinal interface in the region of interest. We found IBA1^+^ (a non-discriminatory macrophage marker) cells in similar regions as cell-like structures identified on the en face vitreoretinal interface slab (arrows, Fig. 1B, E). Interestingly, both highly ramified (yellow arrows) and less dendriform (red arrows) IBA1^+^ cells were detected.

Based upon our findings of macrophage heterogeneity near the retinal surface, we hypothesized that these cells could include microglia, perivascular macrophages, and/or monocyte-derived macrophages. It is well established that microglia [19] and perivascular macrophages [24] are long-lived, self-renewing cells as opposed to monocyte-derived macrophages. We performed fate mapping studies using *Cx3cr1*^*CreER/*+^; *Rosa26*^*zsGreen/*+^ (Mac^GFP^) mice to determine if retinal surface macrophages are blood-derived or self-renewing cells. Mac^GFP^ mice were intraperitoneally injected with tamoxifen to label macrophages GFP^+^ and killed at Week 1 or Week 4 to assess macrophage turnover using retinal sections for optimal determination of the retinal surface. At Week 1, macrophages in the plexiform layer were 97.8% GFP^+^ (Fig. 2A–C, G), consistent with known high labeling of microglia in this model [22]. Alternatively, retinal surface macrophages were only 83.5% GFP^+^ (Fig. 2A–C, red arrows GFP^+^, yellow arrows GFP^neg^) at Week 1 (*p* < 0.05 vs plexiform macrophages, Fig. 2G), suggesting worse labeling efficiency compared to microglia. At Week 4, surface macrophages were 82.4% GFP^+^, which was unchanged from Week 1 (Fig. 2D–G). Similarly, plexiform layer microglia were 95.3% GFP^+^, also unchanged from Week 1. These data show that surface macrophages are consistently 82–83% GFP^+^ at Week 1 and Week 4, suggesting that vitreoretinal interface macrophages are not replenished from the peripheral monocyte pool at steady state.

Since macrophages near the retinal surface are self-renewing cells, we hypothesized that at least a portion of these cells are microglia. Using Mac^GFP^ mice to label macrophages GFP^+^, we investigated what proportion of surface macrophages also expressed the microglia-specific marker TMEM119 [25] using frozen sections. We additionally stained for ICAM2 to investigate localization with retinal vasculature. In the plexiform layers, we found that > 95% of macrophages were GFP^+^TMEM119^+^ and 1% of macrophages were GFP^+^TMEM119^neg^ICAM2^adjacent^ (*p* < 0.001, Fig. 3D). These data show that plexiform layer macrophages were almost entirely microglia, demonstrating the reproducibility of TMEM119 as a microglia marker. At the retinal surface, however, we found that macrophages were 72.3% GFP^+^TMEM119^+^ and 21.2% GFP^+^TMEM119^neg^ICAM2^adjacent^ (*p* < 0.001, Fig. 3D). These results suggest that macrophages near the vitreoretinal interface at steady state are predominantly microglia, with a lesser population of potential perivascular macrophages and possibly a third subtype.

**Fig. 4:**
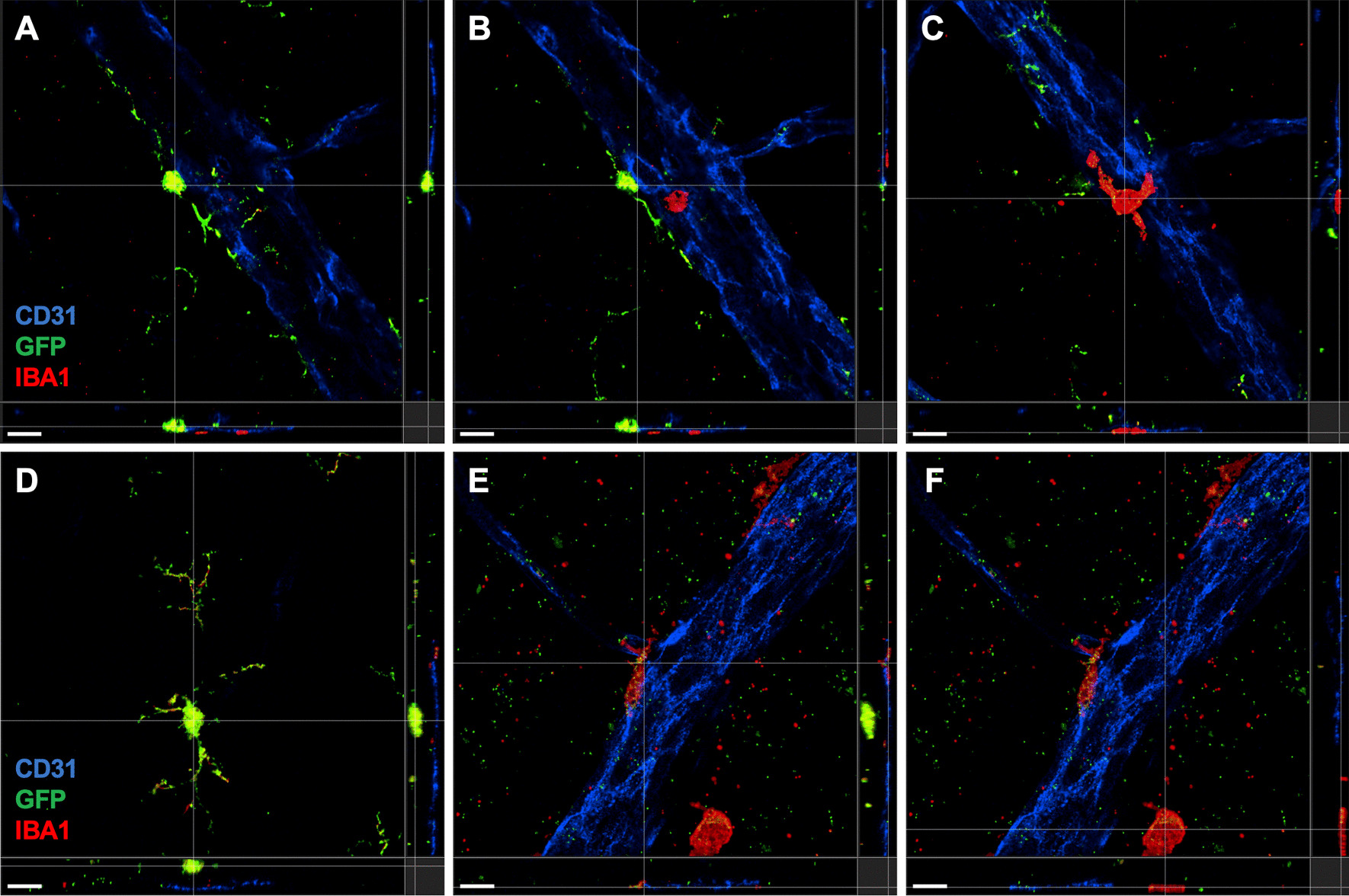
Perivascular macrophages are discrete from microglia. High resolution confocal immunofluorescence of CD31 (blue), GFP (green), and IBA1 (red) from *Tmem119*^*GFP/*+^ retinal flatmounts. For all panels, the main image is the *XY* for a *Z*-plane defined by the gray line in both the YZ plane to the right of the main image and the XZ plane to the bottom. **A**–**C** Z-stack series from just below the retinal surface (**A**) to the superficial level (**C**) demonstrated the difference in ramifications between microglia (yellow/green, **A**, **B**) and perivascular macrophages (red, **C**). The second identically stained series (**D**–**F**) showed the distinction between deeper microglia (green/yellow, **D**), perivascular macrophages (red, adjacent to blood vessel, **E**), and a third macrophage on the bottom right away from the blood vessel (red, **F**). Scale bars are 10 microns. Representative image from 3 independently imaged mice from a single litter

To investigate these macrophage subtypes in more detail, we performed high magnification confocal immunofluorescence of retinal flatmounts with Z-stacks and 3D reconstructions from *Tmem119*^*GFP/*+^ mice. Retinas were stained for GFP, IBA1, and the vascular marker CD31. We detected IBA1^+^GFP^+^ highly ramified microglia (Fig. 4A, B) within 5–10 microns of the retinal surface in close proximity to retinal vessels. In addition, we were able to identify an IBA1^+^GFP^neg^ less ramified putative perivascular macrophage adjacent to the microglia, closer to the vitreoretinal interface, and directly in contact with a large retinal vessel (Fig. 4C). Please see Additional file 1: Video S1, Additional file 2: Video S2, Additional file 3: Video S3 for the full Z-stack and 3 reconstructions of this image. In addition, we found that IBA1^+^GFP^+^ highly ramified microglia in the retinal nerve fiber layer (Fig. 4D) were clearly distinct from a more superficial IBA1^+^GFP^neg^ putative perivascular macrophages (Fig. 4E) and a third IBA1^+^GFP^neg^ non-perivascular macrophage subtype even closer to or on the retinal surface (Fig. 4F). Full Z-stack (Additional file 4: Video S4) and 3D reconstruction (Additional file 5: Video S5, Additional file 6: Video S6) videos further highlight the distinct nature and localization of each of these three cell types.

**Fig. 5:**
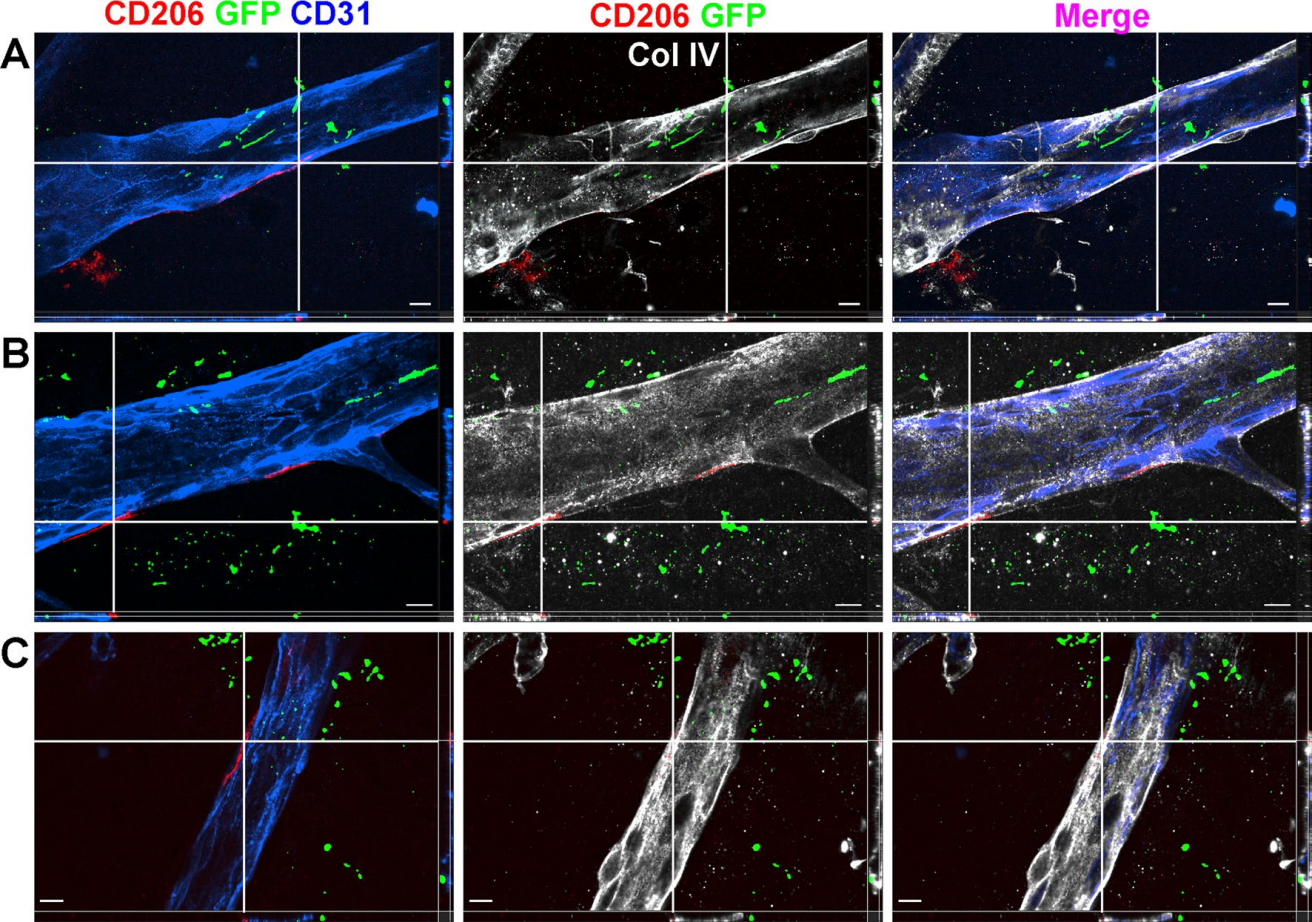
Perivascular macrophage are CD206^+^ and exist within the Collagen IV vascular sheath. High resolution confocal immunofluorescence of CD31 (blue), GFP (green), CD206 (red), and Collagen IV (Col IV, white) from *Tmem119*^*GFP/*+^ retinal flatmounts. For all panels, the main image is the *XY* for a *Z-plane* defined by the thin white line in both the YZ plane to the right of the main image and the XZ plane to the bottom. At each white cross hair, a CD206^+^GFP^neg^ macrophage is adjacent to a CD31^+^ superficial large retinal vessel within 5–10 microns of the retinal surface and within the Collagen IV^+^ vascular sheath. Scale bars are 10 microns. **A**–**C** Are representative images from 3 independently imaged mice from a single litter

To confirm the identity of our putative perivascular macrophage population, we stained whole retinal flatmounts from *Tmem119*^*GFP/*+^ mice with collagen IV and CD206. Collagen IV is a key component of the vascular sheath, which includes basement membranes from both endothelial cells on the luminal side and glial cells from the parenchyma [20]. CD206 is a macrophage marker expressed by perivascular macrophages, but absent in microglia [26]. Similar to Fig. 4, we performed high magnification confocal immunofluorescence of retinal flatmounts in close proximity to the retinal surface with Z-stacks and 3D reconstructions from *Tmem119*^*GFP/*+^ mice. We were able to detect CD206^+^GFP^neg^ cells adjacent to large CD31^+^ vessels at the retinal surface and within the collagen IV perivascular sheath (Fig. 5A–C). These data confirm the presence of perivascular macrophages within 5–10 microns of the true vitreoretinal interface.

**Fig. 6:**
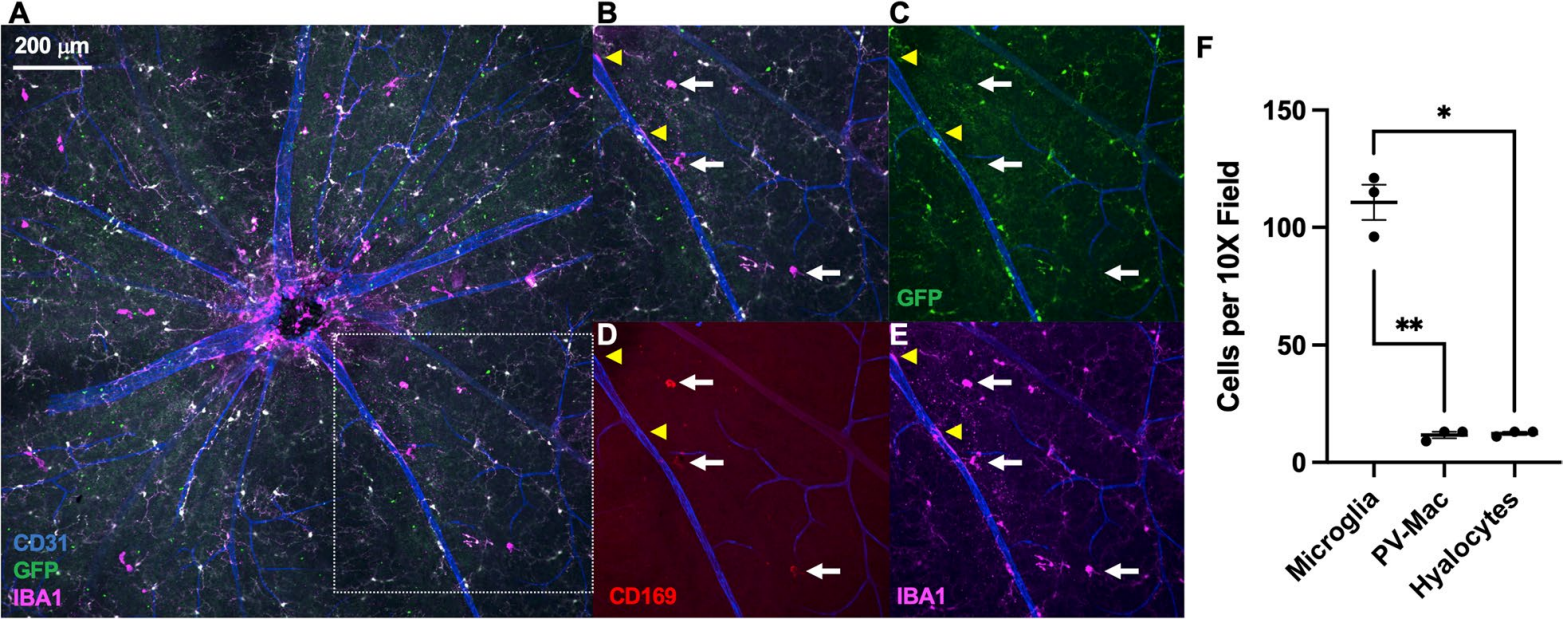
Vitreoretinal interface macrophages include mostly microglia with minor numbers of perivascular macrophages and hyalocytes. **A** Confocal immunofluorescence merged image with CD31 (blue), GFP (green), IBA1 (magenta), and CD169 (red) of a *Tmem119*^*GFP/*+^ retinal flatmount centered at the optic nerve. The highlighted box shows the area magnified in **B**–**E**. Magnified merged (**B**), GFP/CD31 (**C**), CD169/CD31 (**D**), and IBA1/CD31 (**E**) image. White arrows highlight IBA1^+^GFP^neg^CD169^+^ hyalocytes and the yellow arrowheads highlight IBA1^+^GFP^neg^CD169^neg^ perivascular macrophages (PV-Mac). Retinal surface macrophages are composed of 82% IBA1^+^GFP^+^CD169^neg^ microglia, 8% IBA1^+^GFP^neg^CD169^neg^ perivascular macrophages, and 8% IBA1^+^GFP^neg^CD169^+^ hyalocytes (*F*, *p < 0.05, **p < 0.01, repeated measures analysis of variance (ANOVA) followed by Tukey’s multiple comparisons test). *N* = 3 mice from a single litter that were quantitated. The experiment was repeated independently but not quantitated

Vitreal hyalocytes are resident macrophages of the vitreous, detectable at the vitreoretinal interface, and known to express the CD169 cell surface marker [18]. To determine if vitreal hyalocytes are our third steady-state macrophage cell type, confocal immunofluorescence imaging was performed on *Tmem119*^*GFP/*+^ retinal flatmounts at the retinal surface. We quantified IBA1^+^GFP^+^CD169^neg^ microglia (Fig. 6A-C), IBA1^+^GFP^neg^CD169^neg^CD31^adjacent^ perivascular macrophages (Fig. 6B-E, yellow arrowheads), and IBA1^+^GFP^neg^CD169^+^ vitreal hyalocytes (Fig. 6D, white arrows). The retinal surface comprised 82.1% microglia, 8.7% perivascular macrophages, and 9.2% hyalocytes at steady state (Fig. 6F).

**Fig. 7:**
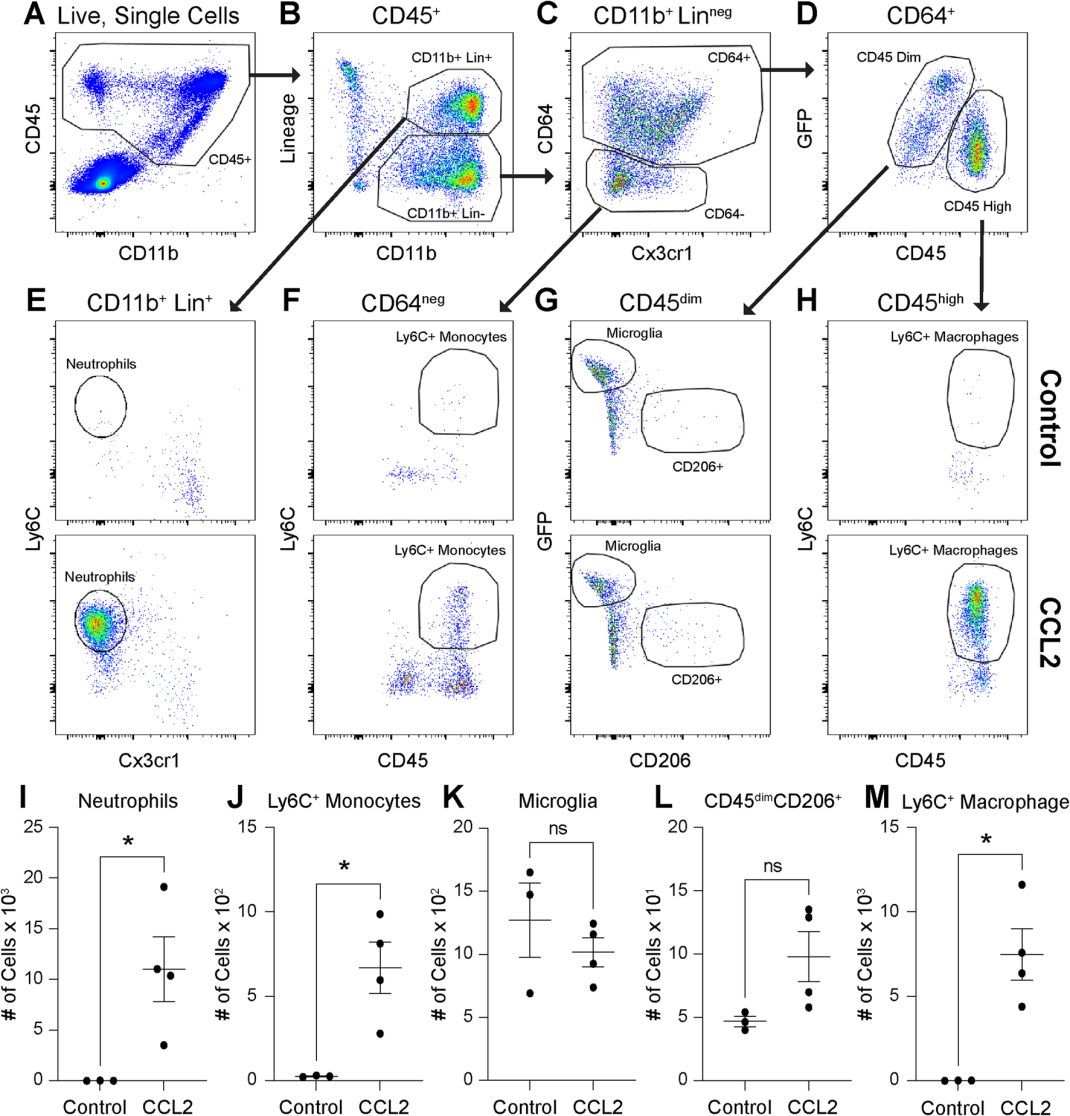
CCL2 injections increase inflammatory cells in the retina. The gating strategy for whole retina flow cytometry. All leukocytes are identified from live, single cells as CD45^+^ (**A**). CD45 + cells are stained with CD11b and Lineage (Lin) gate including CD4 and CD8 (T-cells), B220 (B-cells), NK1.1 (NK cells), SiglecF (eosinophils), Ly6G (neutrophils) to define CD11b^+^Lin^+^ and CD11b^+^Lin^neg^ cells (**B**). CD11b^+^Lin^neg^ cells were gated forward to delineate CD64^+^ and CD64^neg^ cells (**C**). From CD64^+^ cells, CD45^dim^ and CD45^high^ cells were discriminated (**D**). Representative plots of CD11b^+^Lin^+^ cells from control and CCL2 groups identified Ly6C^+^Cx3cr1^neg^ neutrophils (**E**). Representative plots of CD64^neg^ cells from control and CCL2 groups defined Ly6C^+^CD45^+^ classical monocytes (**F**). Representative plots of CD45^dim^ cells from control and CCL2 groups delineated GFP^+^CD206^neg^ microglia and GFP^neg^CD206^+^ resident macrophages (**G**). Representative plots of CD45^high^ cells from control and CCL2 groups discriminated Ly6C^+^ macrophages (**H**). CCL2 treatment increased neutrophil (**I**), classical monocyte (**J**), and Ly6C^+^ macrophage infiltration into the retina (**M**) with no change in microglia or GFP^neg^CD206^+^ macrophages (**K**–**L**). *N* = 3–4 mice (both eyes) per group from 2 independent experiments that were performed on two separate days from different litters and combined, *p < 0.05, two-tailed unpaired t tests with Welch’s correction

Monocyte chemoattractant protein-1 (MCP-1 or CCL2) is the principal ligand for the CCR2 receptor expressed on monocytes and is consistently increased in the eyes of patients with DR and DME [27]. Since macrophage-like cell numbers are increased in PDR [15], we modeled DR and neuroinflammation with intravitreal CCL2 injections. Using multi-parameter flow cytometry of mouse *Tmem119*^*GFP/*+^ retinas, we identified all CD45^+^ leukocytes from live, single cells (Fig. 7A). Next, we used a Lineage gate including Ly6G (neutrophils), SiglecF (eosinophils), CD4/8 (T-cells), B220 (B-cells), and NK1.1 (NK cells) to discriminate Lineage^+^CD11b^+^ and Lineage^neg^CD11b^+^ mononuclear phagocytes (Fig. 7B). From the Lineage^+^CD11b^+^ population, which included eosinophils, neutrophils, and NK1.1 cells, we detected Ly6C^+^Cx3cr1^neg^ neutrophils (Fig. 7E). The Lineage^neg^CD11b^+^ mononuclear phagocytes were gated forward and stained for CD64 to differentiate macrophages from non-macrophage populations (Fig. 7C). From CD64^neg^ cells, we delineated Ly6C^+^CD45^high^ classical monocytes (Fig. 7F). The CD64^+^ macrophage population was separated into CD45^dim^ tissue resident macrophages and CD45^high^ infiltrating macrophages (Fig. 7D). From CD64^+^CD45^dim^ macrophages, we identified GFP^+^CD206^neg^ microglia and GFP^neg^CD206^+^ non-microglia, likely including hyalocytes and perivascular macrophages (Fig. 7G). Finally, CD45^high^ cells were stained with Ly6C to detect Ly6C^+^ tissue infiltrating macrophages (Fig. 7H). In whole retina, we found that neutrophils (590-fold, *p* < 0.05, Fig. 7I), Ly6C^+^ monocytes (24-fold, *p* < 0.05, Fig. 7J), and Ly6C^+^ macrophages (411-fold, *p* < 0.05, Fig. 7M) were significantly increased by CCL2 injections. Alternatively, microglia and CD45^dim^GFP^neg^CD206^+^ non-microglia were unchanged by CCL2 treatment (Fig. 7K, L). These data suggest that CCL2 intravitreal injections mimic neuroinflammation.

**Fig. 8:**
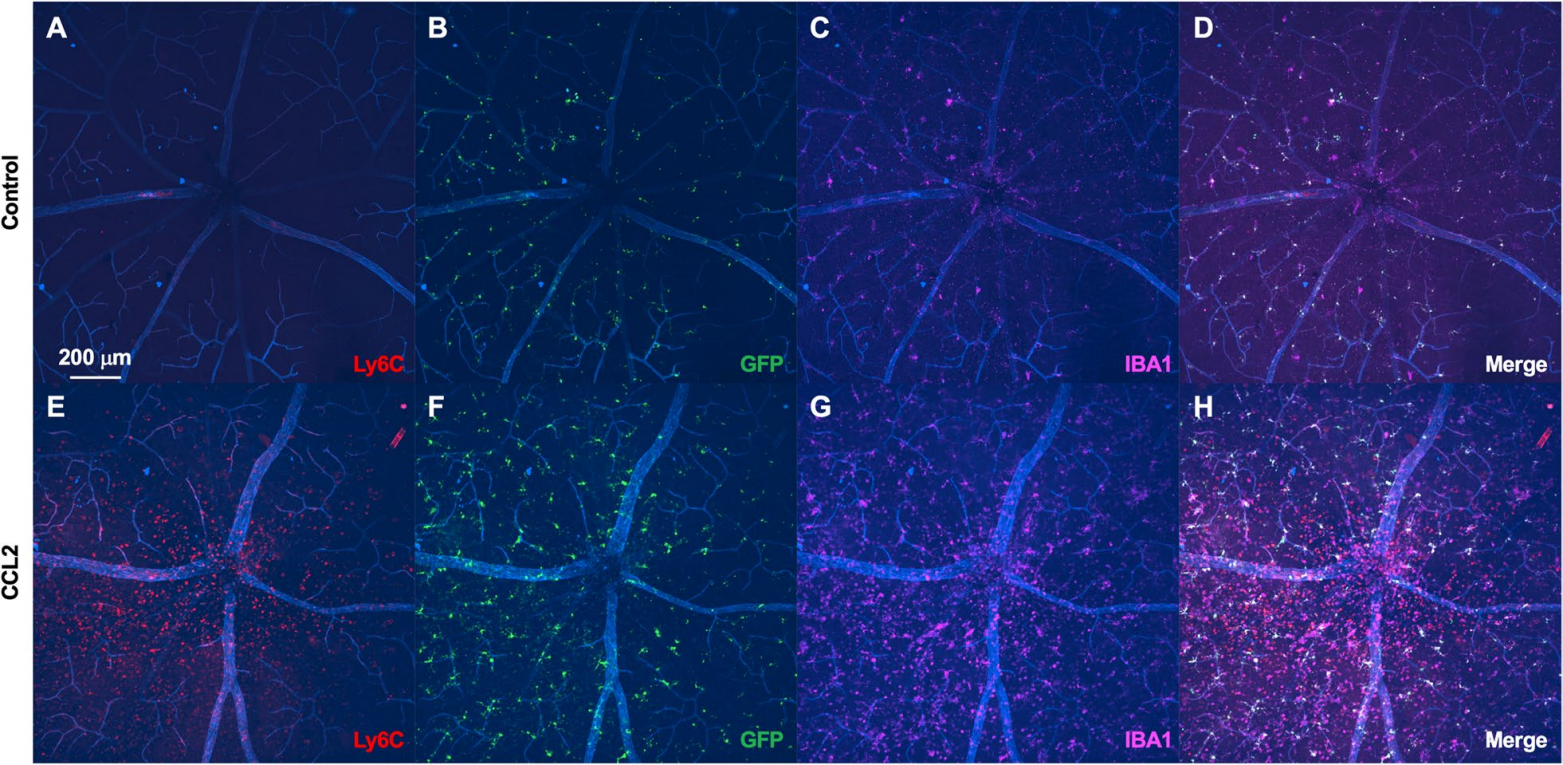
CCL2 injection increased Ly6C^+^ inflammatory cells on the retinal surface. Superficial confocal immunofluorescence images of *Tmem119*^*GFP/*+^ retinal flatmounts stained with CD31 (blue), Ly6C (red), GFP (green), and IBA1 (magenta). Representative image from control retina (**A**–**D**) showed no Ly6C^+^ cells. Representative CCL2-treated mouse demonstrated a dramatic increase in Ly6C^+^ cells at the retina surface (**E**–**H**). Representative image from 3 control and 3 CCL2-treated mice from a single experiment from 2 litters of mice

**Fig. 9:**
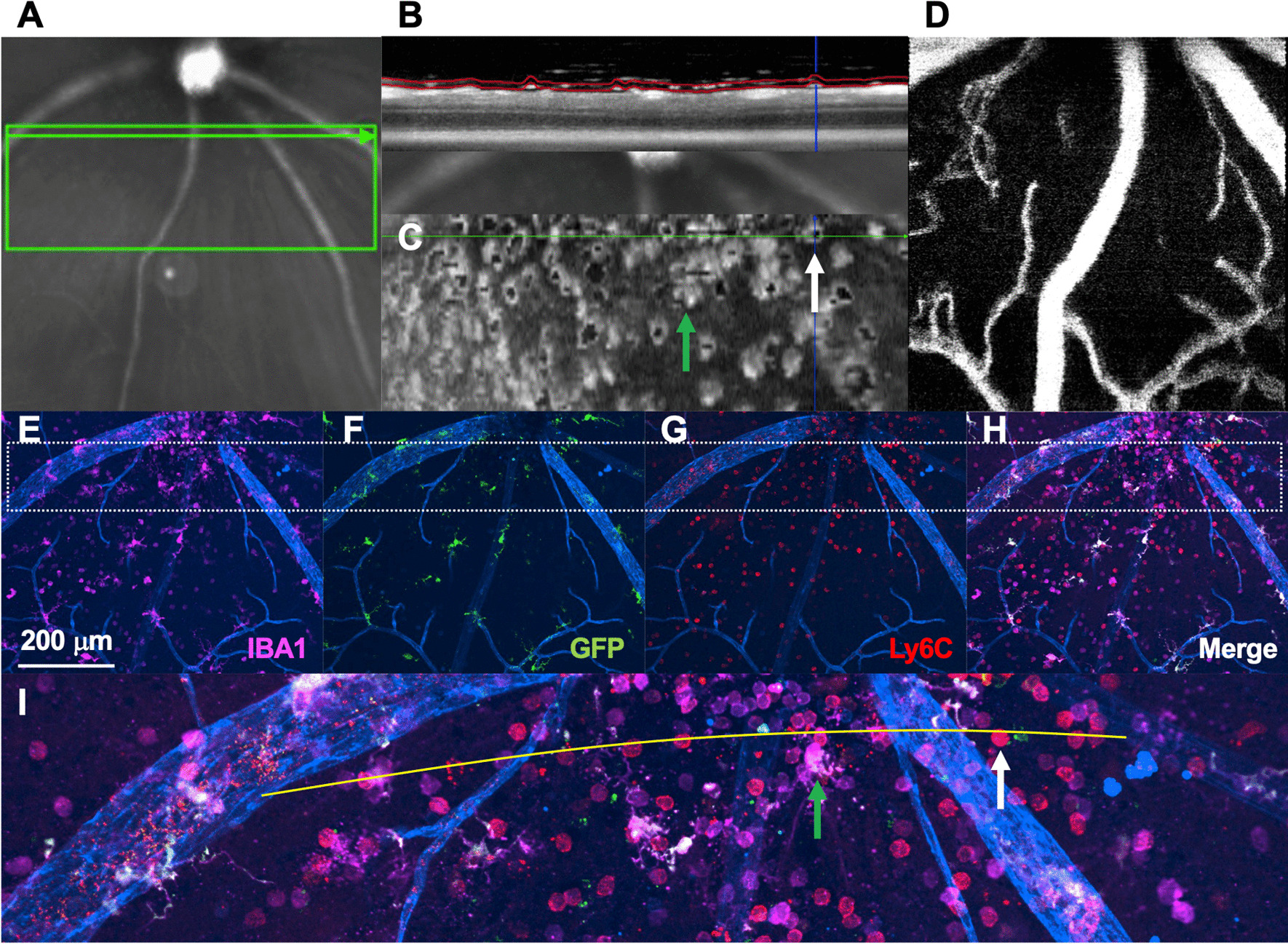
Ly6C^+^ cells are a large portion of vitreoretinal interface macrophages after CCL2 injections. **A** OCT scan location is shown by infrared reflectance. **B** OCT B-scan displaying segmentation of the vitreoretinal interface slab. **C** *En face* vitreoretinal interface slab. Specific cells from **I** are highlighted with arrows. **D** OCT-angiography image. IBA1 (**E**), GFP (**F**), Ly6C (**G**), and merged (**H**) confocal immunofluorescence image of the retinal surface. Dashed box shows the location of the enlarged image in **I**. **I** Enlarged image identified a Ly6C^+^ (white arrow) and an IBA1^+^GFP^+^Ly6C^neg^ cell (green arrow). The curved yellow line in **I** corresponds to the green arrowhead line in **A**. **A** is imaged on the curved inside surface of a live eye, while **I** shows a fixed flatmount. Representative image from three CCL2-treated mice from a single experiment from 1 litter

To investigate the retinal surface, we performed confocal immunofluorescence of retinal flatmounts from *Tmem119*^*GFP/*+^ mice with and without CCL2 injection. Similar to the whole retina, we found no Ly6C^+^ cells at steady state but a highly significant influx of Ly6C^+^ cells near the retinal surface after CCL2 injection (Fig. 8). These data confirm that neuroinflammation after CCL2 treatment includes the retinal surface in addition to the parenchyma. Finally, to examine the vitreoretinal interface, we performed OCT, OCT-angiography, and confocal immunofluorescence of *Tmem119*^*GFP/*+^ mice after CCL2 injections. We found many cells present at the vitreoretinal interface after CCL2 injections using en face OCT analysis just above the vitreoretinal interface (Fig. 9B, C). Each gray circle/ellipse is a cell on the surface of the retina (Fig. 9C). Confocal immunofluorescence imaging confirmed that many of these cells were Ly6C^+^ (white arrow, Fig. 9C, I), but IBA1^+^GFP^+^Ly6C^neg^ microglia were also found (green arrow, Fig. 9C, I). These data suggest that human OCT imaging of macrophage-like cells includes blood-derived inflammatory cells in addition to microglia, perivascular macrophages, and vitreal hyalocytes in the context of CCL2-driven inflammation.

## Discussion

Diabetic retinopathy and RVO are both inflammatory diseases, but current anti-VEGF therapy only targets the vascular component. Thus, identification of inflammatory biomarkers is an important step toward personalized medicine. We and others have found that vitreoretinal interface macrophage-like cell numbers are increased in patients with DR and RVO [15, 16]. However, the identity of vitreoretinal interface macrophage-like cells in these patients is unknown. In this study, we investigated all macrophage subtypes in close proximity to the vitreoretinal interface, including retinal and vitreous cells, because clinical OCT has an axial resolution of only 5–10 microns. We found that vitreoretinal interface macrophages are detectable in mice (Fig. 1), are self-renewing cells (Fig. 2), and comprised predominantly microglia (Fig. 3) with minor populations of perivascular macrophages and vitreal hyalocytes (Figs. 4, 5, 6) during steady state. In an inflammatory context, retinal surface macrophages also include significant numbers of Ly6C^+^ inflammatory cells (Figs. 8, 9). These data suggest that human imaging of macrophage-like cells is indeed a potential biomarker for inflammation in retinal vascular diseases.

At steady state, microglia are the most abundant macrophage subtype within 5–10 microns of the true vitreoretinal interface. Microglia are known to exist in the inner and outer plexiform layers of the retina with distinct layer-based functions [28]. Our data add to this paradigm and suggests that a third retinal nerve fiber layer microglia subset exists and the potential for an independent niche for this microglia subtype is possible. Retinal nerve fiber layer microglia might be increased in patients with glaucoma [12] and PDR [15] because of retinal nerve fiber layer loss that improves visualization of these microglia. Since microglia dysregulation promotes DR progression [7], our identification of microglia as a component of the retinal surface macrophage population is an important finding. However, high powered adaptive optics imaging is necessary to discriminate microglia from other vitreoretinal interface macrophage subtypes in humans. In fact, a recent study using AO-SLO, which has poor axial resolution, tracked vitreoretinal interface macrophage cells and found that the majority of these cells traveled very short distances, staying within their niche [29], suggestive of microglia properties in the parenchyma. Furthermore, our identification of microglia in close proximity to the true vitreoretinal interface suggests that human imaging of macrophage-like cells could be a biomarker for microglia-driven diseases like multiple sclerosis [26].

Perivascular macrophages are a minor population of macrophages near the vitreoretinal interface at steady state. The specific function of perivascular macrophages in DR and RVO is unknown. In the central nervous system, perivascular macrophages play potential roles in blood retinal barrier maintenance [30], vascular permeability, immune cell infiltration [31], neurovascular dysfunction from hypertension [20], and clearance of debris from the perivascular space in the brain [26]. Given the importance of immune cell infiltration and vascular permeability to DR, RVO, and macular edema, the increased numbers of macrophage-like cells at the vitreoretinal interface in patients could include expanded perivascular macrophage populations. Since perivascular macrophages are under-investigated in retinal vascular disease, future studies are warranted, including high resolution transmission electron microscopy, to investigate if perivascular macrophages play potentially a reparative role for endothelial cell damage or a pathogenic role promoting inflammatory cell recruitment and/or vascular permeability. In addition, our identification of perivascular macrophages near the vitreoretinal interface could be applied to diseases like Alzheimer’s where perivascular macrophages clear amyloid debris [32].

Vitreal hyalocytes are also a minor population of macrophages at the retinal surface. Our confocal immunofluorescence findings are highly similar to a prior study differentiating less ramified IBA1^+^F4/80^+^CD169^+^ hyalocytes from dendriform IBA1^+^F4/80^neg^CD169^neg^ microglia at the retinal surface [18]. Human imaging with adaptive optics finds that a minority of retinal surface macrophages are highly mobile and less ramified [29], suggesting that this subpopulation could be hyalocytes. Hyalocyte functions include extracellular matrix production, vitreous immune deviation, modulation of inflammation [33], and regression of the hyaloidal vasculature [34]. Hyalocytes can engulf pigment, and expand with age and during diabetes [18]. Transcriptional profiling of human hyalocytes finds that these cells present antigens and participate in immune privilege of the vitreous [35]. Compared to retinal microglia, human hyalocytes were enriched for angiogenesis and chemotaxis gene ontology terms, suggesting a potential role for hyalocytes in the inflammatory component of DR and RVO [36]. In addition, both diseases can cause vision loss from tractional retinal detachments that form as neovascular membranes contract at the vitreoretinal interface. Macrophages are found in these surgically excised tractional membranes [37, 38] and hyalocytes could be a pathogenic component. Future studies are needed to uncover the role of hyalocytes in retinal vascular diseases.

During inflammation, Ly6C^+^ cells are a significant component of the vitreoretinal interface macrophage population. Classical monocytes are known to promote DR progression in mice [6]. MCP-1 (CCL2) is consistently increased in aqueous and vitreous samples from patients with DR and correlates with macular edema [27]. Furthermore, patients who respond poorly to anti-VEGF have the greatest intraocular MCP-1 and least intraocular VEGF levels [39]. Since MCP-1 is the ligand for CCR2, which is expressed on CCR2^+^Ly6C^+^ classical monocytes, there is a strong link between monocytes and monocyte-derived macrophages in DR pathogenesis and the inflammatory component of treatment-resistant DME. Based on these data, increased macrophage-like cells in patients with advanced DR likely include monocytes and monocyte-derived macrophages, suggesting that retinal surface macrophages are a biomarker for inflammation. Since 32–66% of patients with DME demonstrate persistent retinal thickening despite monthly anti-VEGF treatment [5] and steroids are effective to treat DME [10], using retinal surface macrophage imaging as an inflammatory biomarker could lead to early switch to steroids, improved outcomes, and thus personalized medicine.

## Conclusions

In summary, we find that the retinal surface includes a heterogeneous macrophage population within 5–10 microns of the true vitreoretinal interface. At steady state, most retinal surface macrophages are microglia with a minor population of perivascular macrophages and vitreal hyalocytes. During inflammation, Ly6C^+^ cells are a significant additional component to the heterogenous vitreoretinal interface macrophage population. Additional human and animal studies are needed on this emerging inflammatory biomarker field. These findings expand our knowledge of the vitreoretinal interface and have wide implications from retinal vascular disease to inflammatory and degenerative central nervous system conditions.

## Supplementary Information

**Additional file 1: Video S1.** Z-stack video 1. Z-stack video from Fig. 4A–C.

**Additional file 2: Video S2.** 3D reconstruction 1. 3D reconstruction from Fig. 4A–C.

**Additional file 3: Video S3.** 3D reconstruction 1 with expanded Z axis. 3D reconstruction from Fig. 4A–C with Z axis expanded by 2X to enhance visualization.

**Additional file 4: Video S4.** Z-stack video 2. Z-stack video from Fig. 4D–F.

**Additional file 5: Video S5.** 3D reconstruction 2. 3D reconstruction from Fig. 4D–F.

**Additional file 6: Video S6.**

## Data Availability

The datasets used and analyzed for mouse studies are available from the corresponding author on reasonable request.

## Abbreviations

DR: Diabetic retinopathy
RVO: Retinal vein occlusion
VEGF: Vascular endothelial growth factor
DME: Diabetic macular edema
OCT: Optical coherence tomography
PDR: Proliferative diabetic retinopathy
ANOVA: Analysis of variance
*Cx3cr1*^CreER/+^; *Rosa26*^zsGreen/+^: Mac^GFP ^GFP: Green fluorescent protein
MCP-1 or CCL2: Monocyte chemoattractant protein-1
PV-Mac: Perivascular macrophages
ILM: Internal limiting membrane
AO-SLO: Adaptive optics scanning laser ophthalmoscopy
